# Dendritic Cells Pulsed with Cytokine-Adjuvanted Tumor Membrane Vesicles Inhibit Tumor Growth in HER2-Positive and Triple Negative Breast Cancer Models

**DOI:** 10.3390/ijms22168377

**Published:** 2021-08-04

**Authors:** Luis E. Munoz, Lenore Monterroza, Ramireddy Bommireddy, Yalda Shafizadeh, Christopher D. Pack, Sampath Ramachandiran, Shaker J. C. Reddy, Periasamy Selvaraj

**Affiliations:** 1 Department of Pathology and Laboratory Medicine, Emory University School of Medicine, Atlanta, GA 30322, USA; luis.munoz@emory.edu (L.E.M.); lenore.monterroza@emory.edu (L.M.); rbommi2@emory.edu (R.B.); yalda.ma89@gmail.com (Y.S.); 2 Metaclipse Therapeutics Corporation, Atlanta, GA 30340, USA; cpack@metaclipse.com (C.D.P.); sramachandiran@metaclipse.com (S.R.); sreddy@metaclipse.com (S.J.C.R.); 3 Department of Pathology and Laboratory Medicine, Emory University, Woodruff Memorial Building, Room 7309 101 Woodruff Circle, Atlanta, GA 30322, USA

**Keywords:** immunotherapy, vaccine, breast cancer, dendritic cells, HER2, GPI-IL-12, GPI-GM-CSF

## Abstract

Dendritic cells (DCs) are the most effective antigen presenting cells for the development of T cell responses. The only FDA approved DC-based immunotherapy to date is Sipuleucel-T, which utilizes a fusion protein to stimulate DCs ex vivo with GM-CSF and simultaneously deliver the antigen PAP for prostate cancer. This approach is restricted by the breadth of immunity elicited to a single antigen, and to cancers that have a defined tumor associated antigen. Other multi-antigen approaches have been restricted by poor efficacy of vaccine adjuvants. We have developed a vaccine platform that consists of autologous DCs pulsed with cytokine-adjuvanted tumor membrane vesicles (TMVs) made from tumor tissue, that encapsulate the antigenic landscape of individual tumors. Here we test the efficacy of DCs pulsed with TMVs incorporated with glycolipid-anchored immunostimulatory molecules (GPI-ISMs) in HER2-positive and triple negative breast cancer murine models. Pulsing of DCs with TMVs containing GPI-ISMs results in superior uptake of vesicles, DC activation and cytokine production. Adaptive transfer of TMV-pulsed DCs to tumor bearing mice results in the inhibition of tumor growth, reduction in lung metastasis, and an increase in immune cell infiltration into the tumors. These observations suggest that DCs pulsed with TMVs containing GPI-GM-CSF and GPI-IL-12 can be further developed to be used as a personalized immunotherapy platform for cancer treatment.

## 1. Introduction

Dendritic cell (DC)-based immunotherapy approaches have gained popularity for their ability to induce potent anti-tumor immune responses [1]. Currently, the only vaccination approach that is FDA approved for cancer is the DC-based vaccine Sipuleucel-T [2]. This DC-based vaccine relies on obtaining peripheral-blood mononuclear cells (PBMCs) from patients and stimulating ex vivo with a fusion protein consisting of GM-CSF and the tumor antigen prostatic acid phosphatase (PAP). Sipuleucel-T has provided some survival advantage in prostate cancer patients [2]. Despite this success, the breadth of immunity induced by a single antigen approach is limited and not widely applicable to cancers without defined target antigens, such as triple negative breast cancer. Other DC vaccination approaches rely on the identification and production of patient specific neoantigens from peptides that are restricted to the human leukocyte antigen (HLA) class I for presentation to CD8 T cells [3,4,5]. Delivery of mRNA encoding for tumor antigens and activating signals to DCs has also been reported with some success [6]. In one of the first-in-human testing of personalized RNA vaccines, individualized RNAs encoding poly-neo-epitopes were delivered to patients at the inguinal lymph nodes for DC uptake. Therapy with these personalized “mutanome” vaccines resulted in vaccine-induced T cell infiltration into tumors, reduced metastasis, and sustained progression-free survival [7]. However, these approaches remain resource-intensive [8]. GVAX is another notable approach that utilized irradiated tumor cells modified to express soluble GM-CSF to stimulate antigen presenting cells and induce an anti-tumor response [9]. However, the difficulty to regulate the amount of soluble GM-CSF secreted resulted in increased myeloid-derived suppressor cell (MDSC) and impaired DC responses in patients [10]. Tumor lysates have also been tested as source of antigen for DC based vaccines in cancer. Oxidation of tumor tissue using hypochlorous acid was shown to increase the immunogenicity of the lysate. DCs generated from monocytes and pulsed with the autologous oxidized tumor lysate have been shown to induce robust anti-tumor effects in murine and human studies [11,12].

We have developed a tumor membrane vesicle (TMV)-based vaccine immunotherapy approach that can be prepared from frozen tumor tissue. TMVs are 200–600 nm in size, making them ideal for processing by antigen presenting cells [13,14], such as DCs and macrophages. To increase the immune response against the tumor antigens in the TMV, we have developed a novel “protein transfer” approach that incorporates glycosyl phosphatidylinositol (GPI)-anchored immunostimulatory molecules (GPI-ISMs) onto the surface of TMVs. In previous studies we have utilized TMVs incorporated with GPI-B7-1 and GPI-IL-12 to stimulate the anti-tumor response in preclinical models of breast cancer [15,16], thymoma [17], and head-and-neck cancers [18]. While these approaches are promising when the tumor tissue is available in sufficient quantity, it is difficult to generate sufficient TMV vaccine for the cancers where the tissue is limited in quantity, such as inoperable tumors or metastatic recurrent cases where the archived primary tumor is no longer available at the time of recurrence where only a biopsy sample can be used. To overcome this caveat, developing a DC vaccine by delivering a lower dose of TMVs to antigen presenting cells ex vivo to enhance the TMV uptake, DC activation and anti-tumor immune response in a therapeutic setting will be a promising approach.

In this study, we have developed a DC vaccine platform that utilizes autologous DCs pulsed with cytokine-adjuvanted TMVs as a therapeutic in murine breast cancer models. We observed that DCs can take up TMVs when pulsed in vitro, and this uptake is improved when TMVs contain GPI-GM-CSF and GPI-IL-12 on their surface. TMVs containing GPI-GM-CSF and GPI-IL-12 can also improve DC activation as measured by surface CD86 and MHC-II expression as well as cytokine production. We also show that treatment with DCs pulsed ex vivo with HER-2+ TMV results in tumor growth inhibition and improved survival of tumor bearing mice. Further, in a triple negative breast cancer model which lacks a defined target antigen, the DC vaccine can also inhibit tumor growth, reduce lung metastasis and increase immune cell infiltration into the tumor.

## 2. Materials and Methods

### 2.1. Mice

BALB/c mice (6–8-week-old female) purchased from Jackson Laboratories (Bar Harbor, ME, USA) were housed in the Division of Animal Resources facility. Five mice were used per group as described in the figure legends. All animal experiments were done in accordance with the Emory University Institutional Animal Care and Use Committee (IACUC) approved protocols (DAR-2017-00-504). Female mice were selected to match the sex of origin from 4T1 and D2F2/E2 cell lines.

### 2.2. Cell Lines

The TNBC cell line 4T1 (CRL-2539) was obtained from ATCC. The D2F2/E2 cell line was a kind gift from Dr. Wei-Zen Wei from Wayne State University. D2F2/E2 cells were generated by transfecting D2F2 cells with full length human ErbB-2 (HER2) [19]. Cell lines were verified using their expression profile of surface markers and morphology. Mycoplasma testing was performed routinely as a precautionary quality check. These cell lines were cultured in complete DMEM with 10% Hyclone FBS (Cytiva, Marlborough, MA, USA), 4500 mg/L glucose (MilliporeSigma, Burlington, MA, USA), Gibco Glutamax (Thermo Fisher Scientific, Waltham, MA, USA), and Penicillin-Streptomycin (MilliporeSigma, Burlington, MA, USA). Selection medium for D2F2/E2 cells contained 800 μg/mL G418 (MilliporeSigma, Burlington, MA, USA).

### 2.3. TMV Preparation, Characterization, and GPI-ISM Incorporation

TMVs were generated from frozen tumor tissue or cultured cell pellets as previously described [15,16]. Tumor tissue was homogenized, and the plasma membranes were isolated by ultracentrifugation over a 41% sucrose gradient. TMVs were analyzed for particle diameters using a Zetasizer Nano ZS from Malvern Panalytical (Malvern, UK). The GPI-ISMs GPI-IL-12 and GPI-GM-CSF were incorporated using protein transfer by incubating 25 µg of purified GPI-ISMs per milligram of TMV at 37 °C for 4 h under constant rotation. TMVs with incorporated GPI-ISMs were then pelleted by ultracentrifugation and unbound GPI-ISMs in the supernatant were removed, followed by a wash with PBS. Incorporation of GPI-GM-CSF and GPI-IL-12 was verified via flow cytometry by using anti-mouse GM-CSF PE (clone MP1-22E9) and anti-mouse IL-12 APC (clone C17.8) antibodies.

### 2.4. Bone Marrow-Derived Dendritic Cell (BMDC) Production

BMDCs were generated according to established protocols [20]. Briefly, femurs of female BALB/c mice were removed and cleaned from surrounding muscle tissue. Bone marrow was flushed using RPMI-1640 medium with a 22G needle and syringe. Red blood cells (RBC) were lysed using RBC lysis buffer (MilliporeSigma, Burlington, MA, USA) and resulting cells were cultured in complete RPMI-1640 medium containing 20 ng/mL recombinant murine GM-CSF (rmGM-CSF, BioLegend, San Diego, CA, USA) at a density of 2 × 10^5^ cells/mL. The rmGM-CSF was replenished at days 3, 6 and 8 in culture and BMDCs were ready for use at day 10.

### 2.5. TMV Uptake, DC Pulsing, and Tumor Challenge Studies

For BMDC uptake, TMVs were labeled using the pH sensitive pHrodo Deep Red mammalian and bacterial cell labeling kit (Invitrogen, Waltham, MA, USA) following manufacturers recommendations. Briefly, 50 µg of TMV was diluted in 1 mL of Cell Labeling and Wash buffer with 1.5 µg of pHrodo Deep Red dye. The mixture was incubated for 2 h at room temperature, then washed once with wash buffer, followed by a PBS wash. No fluorescence of TMVs was detected by flow cytometry with FACS buffer (PBS with 5% FBS, 0.05% sodium azide, 5 mM EDTA) at pH 7 but detected only after acidification to pH 4 with diluted HCl (Supplementary Figure S1). The BMDCs were cultured with pHrodo labeled TMVs for 30, 90 or 180 min and followed by flow cytometry analysis. For BMDC pulsing, 400,000 DCs/mL were cultured in 1 mL of media with or without 20 µg of TMVs. Lipopolysaccharide (LPS) was used as a positive control for activation at a concentration of 1 µg/mL. For D2F2/E2 challenge studies, BALB/c mice were inoculated with 2 × 10^5^ D2F2/E2 cells in 100 µL PBS subcutaneously (s.c.) in the hind flank. For 4T1 challenge studies, BALB/c mice were inoculated with 5 × 10^4^ 4T1 cells in 100 µL PBS s.c. in the hind flank. Tumor diameters were measured twice per week using a digital vernier caliper. BMDCs were pulsed with the respective D2F2/E2 or 4T1 TMVs for 24 h, followed by two rinses with PBS to remove unbound TMV. DCs were harvested and washed twice with PBS by centrifugation at 300× *g* for 5 min and injected s.c. into the opposite flank of the tumor at 1 × 10^6^ DCs per dose.2.6. Cytokine ELISA.

BMDCs were treated with TMVs as described above. Cell culture supernatants were collected, and the levels of IFN-γ, IL-6 and TNF-α were determined with the ELISA MAX Standard Set Mouse IFN-γ, IL-6 and TNF-α kits (BioLegend, San Diego, CA, USA). Plates were read on an Epoch microplate reader (BioTek, Winooski, VT, USA) at 450 nm for signal detection and 540 nm for background correction.

### 2.6. 4T1 Lung Metastasis Assay

Lungs were collected from mice bearing 4T1 tumors after 20 days of tumor inoculation. These were processed under sterile conditions, minced, and digested in 1 mg/mL Collagenase IV (MilliporeSigma, Burlington, MA, USA) for 2 h at 37 °C under rotation. The cell suspension was filtered and washed twice in selection media composed of complete DMEM containing 60 µM 6-thioguanine (6-TG). This treatment kills lung fibroblasts without affecting tumor cells. Once one of the wells reached confluency, all the wells were harvested and counted using a Cellometer T4 Auto counter (Nexelcom, Lawrence, MA, USA).

### 2.7. Tumor Infiltrating Cell Staining and Analysis

4T1 tumors were resected, minced, and digested in Liberase TL (Roche, Basel, Switzerland) and DNAse (Roche, Basel, Switzerland) for 30 min at 37 °C under constant rotation. Single cell suspension was filtered through 70 μm strainers and washed with PBS. Cells were then incubated with Live Dead NIR in PBS for 20 min and then Fc blocking antibody (2.4G2, Biolegend, San Diego, CA, USA) in FACS buffer at 4 °C for 15 min. Fluorochrome-conjugated antibodies specific to cell surface markers were added and incubated for 45 min at 4 °C. Fluorochrome-conjugated antibodies to CD45 (1:500 FITC clone 30-F11), CD3 (1:100 BV785 clone 17A2), CD8 (1:300 APC clone 53-6.7), CD4 (1:300 BB700 clone RM4-5), MHC-II (1:200 BV605 clone M5/114.15.2), and CD11c (1:200 AF700 clone N418) were obtained from BioLegend (San Diego, CA, USA). The stained samples were acquired in an Aurora cytometer (Cytek, Fremont, CA, USA) and data was analyzed using FlowJo version 10.8 (FlowJo LLC, Ashland, OR, USA).

### 2.8. Statistical Analysis

All statistical analyses were performed using Prism version 9.1.2 (GraphPad Software, San Diego, CA, USA). The type of statistical test, *p* value and *n* values are listed in figure legends. One-way ANOVA was used to analyze the differences in cell surface marker expression, cytokine production, and lung metastasis quantification. Two-way ANOVA was used to assess the differences in tumor growth over time. Survival was analyzed using a Log-rank (Mantel-Cox) test to compare treatment groups.

## 3. Results

### 3.1. TMV Vaccine Contain the Tumor Antigen HER2 and Protein Transferred GPI-ISMs on Their Surface

**Figure 1:**
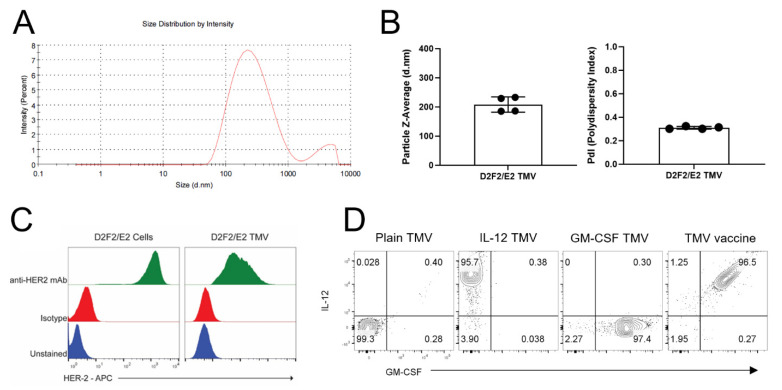
Tumor membrane vesicles vaccine characterization and GPI-ISM incorporation. (**A**), Zetasizer size distribution of TMVs prepared from D2F2/E2 cells. (**B**), Particle Z-Average (left panel) and polydispersity index (right panel) of four runs of D2F2/E2 TMVs on the Zetasizer. Error bars represent SD. (**C**), Surface expression of HER2 antigen on D2F2/E2 cells (left panel) and TMVs prepared from D2F2/E2 cells (right panel). (**D**), Flow cytometry plots of IL-12 and GM-CSF on D2F2/E2 TMVs without GPI-ISMs (Plain TMV), with GPI-IL-12 (IL-12 TMV), GPI-GM-CSF (GM-CSF TMV) and both GPI-ISMs incorporated (TMV vaccine) by protein transfer.

To characterize TMVs, flow cytometry and Zetasizer analysis were used to assess the surface marker expression and size distribution of the vesicles. The data suggest that D2F2/E2 TMVs prepared from cell culture pellets are within 200 nm in diameter (Figure 1A,B). The polydispersity index of the vesicles shows an average measurement of 0.31, which is similar to that of liposome formulations [21]. Surface marker expression of HER2 was performed on D2F2/E2 cells and TMVs from this cell line. D2F2/E2 cells express high levels of HER2 on the surface, and TMVs retain this tumor antigen on their surface (Figure 1C). Flow cytometry analysis of plain TMVs show no signal of IL-12 or GM-CSF on their surface, while analysis of GPI-ISM-incorporated TMV (TMV vaccine) confirms the presence of protein transfer-mediated incorporation of GPI-GM-CSF and GPI-IL-12 (Figure 1D).

### 3.2. Uptake of TMVs by DCs Is Potentiated by GPI-ISMs

**Figure 2:**
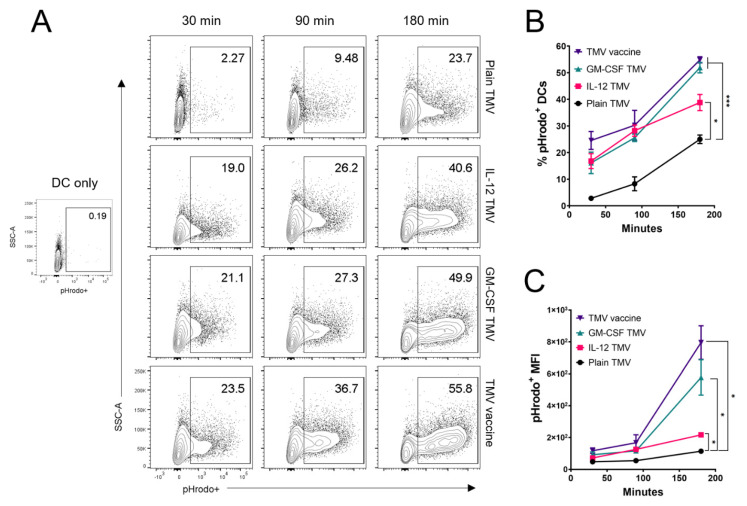
TMV uptake by DCs is potentiated by GPI-ISMs. (**A**), TMVs were labeled with the pH sensitive dye pHrodo Deep Red and incubated with BMDCs for 30, 90 or 180 min, representative contour plots of BMDCs after treatment. (**B**), BMDCs were analyzed by flow cytometry to quantify the percentage of cells that had taken up TMVs into the acidic endolyzosome which activates fluorescence of pHrodo labeled TMVs (*n* = 3). (**C**), measurement of the pHrodo MFI of the BMDCs that had taken up TMVs (*n* = 3). *p* < 0.05 *, *p* < 0.001 ***. Analysis done in (**B**,**C**) with two-way ANOVA with Tukey’s test for multiple comparisons, error bars represent SD.

DCs are one of the most potent APCs that can take up vaccine antigens at the vaccination site and migrate to lymphoid organs to initiate a T cell response [22]. To test whether DCs can phagocytose TMVs incorporated with GPI-ISMs, we labeled TMVs with the pH sensitive dye pHrodo Deep Red, which can fluoresce in the acidic conditions of the late endosome/phagolysosome as described in methods. BMDCs were pulsed with pHrodo labeled TMVs for 30, 90, and 180 min, then washed and kept on ice until flow cytometry analysis. DCs that were pulsed with plain TMV were able to take up TMVs as early as 30 min, albeit at a smaller percentage compared to DCs pulsed with TMV containing either GPI-ISM. After 180 min of incubation, there is a significant increase in the percentage of pHrodo^+^ DCs pulsed with GPI-IL-12 TMVs; however, the highest percentages of pHrodo^+^ DCs were observed after pulsing with GPI-GM-CSF TMVs or TMV vaccine containing both GPI-ISMs (Figure 2A,B). There is also a significant increase in the pHrodo MFI on DCs pulsed with TMVs containing either GPI-IL-12 or GM-CSF compared to plain TMVs; however, DCs pulsed with TMVs containing both GPI-IL-12 and GPI-GM-CSF showed the highest MFI (Figure 2C).

### 3.3. TMVs Incorporated with GPI-ISMs Can Induce DC Activation and Cytokine Production

**Figure 3:**
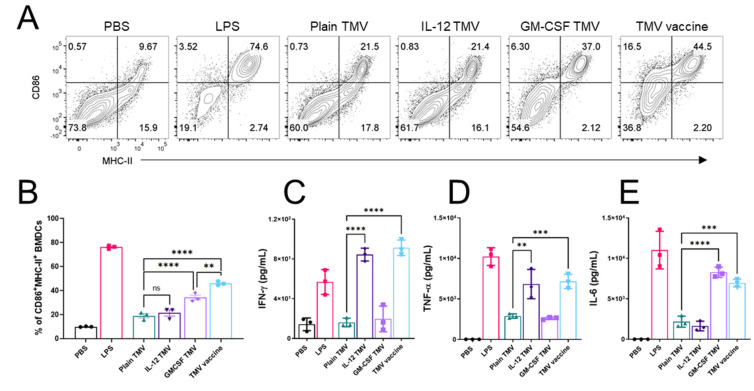
TMVs with GPI-ISMs induce DC activation and cytokine release. (**A**), Representative plots of surface levels of CD86 and MHC-II on BMDCs after stimulating with LPS (positive control), D2F2/E2 TMVs containing different GPI-ISMs, or unstimulated (PBS) for 24 hrs. (**B**), quantification from A percentage of CD86^+^ MHC-II^+^ BMDCs after 24-h pulsing with TMVs. (**C**–**E**), ELISA of IFN-γ, TNF-α, and IL-6 levels in supernatants of BMDCs stimulated as in (**A**). Error bars represent SD. **, *p* < 0.001 ***, *p* < 0.0001 ****, *p* > 0.05 ns. Analysis in (**B**–**E**) done with a one-way ANOVA with Tukey’s post test for multiple comparisons.

Given that DCs were able to take up TMVs *in vitro*, we tested the ability of these cells to upregulate the activation markers CD86 and MHC-II, as well as production of inflammatory cytokines after pulsing with TMVs made from D2F2/E2 cells. DCs pulsed with plain TMVs or TMVs containing GPI-IL-12 showed no significant activation as measured by CD86 and MHC-II expression (Figure 3A,B). DCs that were pulsed with TMVs containing GPI-GM-CSF or both GPI-ISMs showed a significant increase in activation marker expression (Figure 3B). Cytokine production was measured as a proxy to GPI-ISM functionality. DCs pulsed with TMVs containing GPI-IL-12 showed a slight increase in production of IFN-γ compared to plain TMV pulsing (Figure 3C). Similarly, those DCs that were pulsed with GPI-IL-12 TMVs also showed an increase in TNF-α production (Figure 3D). Interestingly, those DCs that were pulsed with GPI-GM-CSF TMVs showed an increased production of IL-6 (Figure 3E) but no increase in IFN-γ or TNF-α. The presence of GPI-GM-CSF did not inhibit or potentiate the production of cytokines induced in the presence of GPI-IL-12, as evidenced by the cytokine production of DCs pulsed with the TMV vaccine containing both GPI-ISMs. 

### 3.4. DC Vaccine Therapy Reduces Tumor Growth

**Figure 4:**
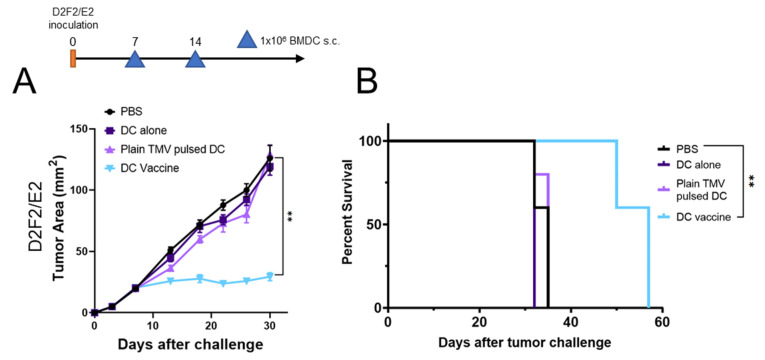
DC vaccine inhibits D2F2/E2 tumor growth and increase the survival of mice. (**A**), BALB/c mice (*n* = 5 per group) were inoculated with 2 × 10^5^ D2F2/E2 cells s.c. on the flank. BMDCs were pulsed for 24 h with D2F2/E2 TMV (Plain TMV pulsed DC), or D2F2/E2 TMV incorporated with GPI-IL-12 and GPI-GM-CSF (DC vaccine) and administered at day 7 and 14 s.c. on the contralateral flank (1 × 10^6^ DCs/dose). (**B**), survival of tumor bearing mice is shown. *p* < 0.01 **. (**A**) was analyzed with a two-way ANOVA with Tukey’s post-test for multiple comparisons, error bars represent SEM. Log-rank (Mantel-Cox) test was used to determine significance in survival of mice.

To test the efficacy of the DCs pulsed with TMVs as a vaccine platform, we used the HER2 positive murine model D2F2/E2. The D2F2/E2 model represents an immunogenic tumor model due to the presence of the human tumor antigen HER2. Mice were challenged with D2F2/E2 cells and BMDCs were delivered s.c. on the opposite flank after seven days of tumor growth. Treatment with DCs that were not pulsed with TMV (DC alone), and DCs that were pulsed with TMVs without GPI-ISMs (plain TMV pulsed DCs) showed no reduction of tumor growth and had similar survival rates compared to PBS controls (Figure 4A,B). However, mice that received the DC vaccine (DCs pulsed with TMVs incorporated with GPI-IL-12 and GPI-GM-CSF) showed a significant inhibition of tumor growth (Figure 4A) compared to all other groups. Mice from the PBS control group, DC alone or Plain TMV pulsed DC groups only survived until day 35 post tumor inoculation. Treatment with the DC vaccine resulted in a significant improvement in survival (day 35 versus day 57) compared to controls (Figure 4B).

### 3.5. DC Vaccine Inhibits 4T1 Tumor Growth, Reduces Lung Metastasis, and Improved Immune Profile of Tumors

**Figure 5:**
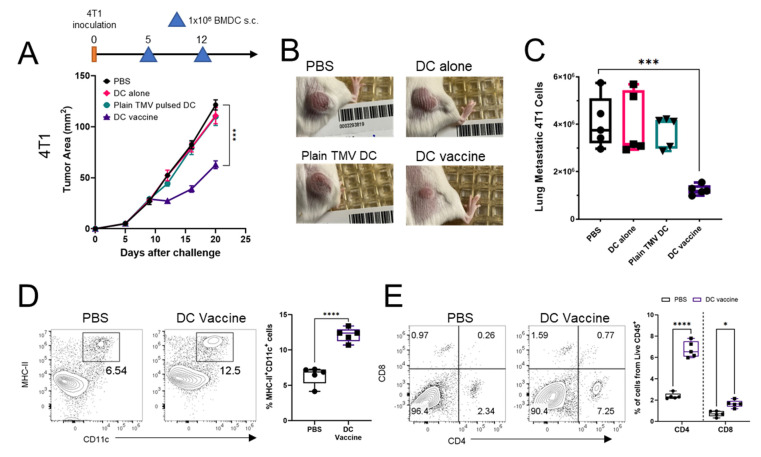
DC vaccine inhibits tumor growth, reduces lung metastasis, and increases immune cell infiltration into 4T1 tumors. (**A**), BALB/c mice (*n* = 5 per group) were inoculated with 5 × 10^4^ 4T1 cells s.c. on the flank. DCs were pulsed for 24 h with 4T1 TMV (Plain TMV pulsed DC), or 4T1 TMV incorporated with GPI-IL-12 and GPI-GM-CSF (DC vaccine) and administered at days 5 and 12 s.c. on the contralateral flank at (1 × 10^6^ DCs/dose). (**B**), pictures of tumors from A at day 20. (**C**), lungs were harvested at day 20 and prepared into single cell suspension then grown in selection media containing 6-thioguanine to quantify metastatic 4T1 cells. (**D**), contour plots and quantification of MHC-II^+^ CD11c^+^ DCs from CD45^+^ cell in 4T1 tumors. (**E**), contour plots and quantification of CD4^+^ and CD8^+^ T cells from CD45^+^ cells in 4T1 tumors. *p* < 0.05 *, *p* < 0.001 ***, *p* < 0.0001 ****. (**A**) was analyzed with a two-way ANOVA with Tukey’s post-test for multiple comparisons, error bars represent SEM. (**C**–**E**) were analyzed with a one-way ANOVA with Tukey’s post-test for multiple comparisons.

The 4T1 TNBC model represents a highly metastatic cancer that lacks a defined target antigen. Mice were challenged with 4T1 cells and DCs pulsed with 4T1 TMV vaccine were injected on the opposite flank after five days of tumor inoculation. Similar to the observations in the D2F2/E2 model, only treatment with DCs pulsed with 4T1 TMVs containing both GPI-ISMs (DC vaccine) resulted in a significant inhibition of tumor growth (Figure 5A,B). Given that the 4T1 model is spontaneously metastatic, the metastatic burden in the lungs of tumor bearing mice was quantified after 20 days of tumor growth. Treatment with the DC vaccine resulted in a 2 to 3-fold reduction in metastatic 4T1 cells in the lungs, compared to PBS controls, DC alone, or plain TMV pulsed DC therapy (Figure 5C). To characterize the immune infiltrates of 4T1 tumors after therapy with TMV vaccine, tumors were resected and processed as described in the methods section to isolate tumor infiltrating cells. Flow cytometry analysis showed a 2-fold increase of MHC-II^+^ CD11c^+^ DCs in 4T1 tumors after mice received the DC vaccine compared to PBS controls (Figure 5D). Further, DC vaccine treatment resulted in a significant increase in CD4 and CD8 T cells in the tumor compared to PBS controls (Figure 5E).

## 4. Discussion

Our studies demonstrate that therapy with DCs as delivery vehicles for the TMV vaccine can inhibit tumor growth in two different breast cancer models. In this approach, DCs are presented simultaneously with tumor antigens and adjuvants (GPI-ISMs) in a particulate form to enhance uptake of the vaccine and activation of DCs. We find that DCs pulsed with TMV vaccine had improved uptake, while GPI-GM-CSF on TMVs induced DC activation, and GPI-GM-CSF and GPI-IL-12 provided signaling to produce inflammatory cytokines. We also show that the TMV vaccine platform can be used in the metastatic 4T1 model, which lacks a defined tumor antigen, where vaccine pulsed DCs can result in the inhibition of tumor growth, reduction of lung metastatic burden, and increased tumor-infiltrating immune cells. 

The optimal delivery of antigens to DCs is one of the most important aspects in vaccine formulations. Particles that are within the 100 nm to 1 μm diameter have shown good immunogenicity and uptake by DCs [13,23,24]. TMVs are derived from plasma membrane of tumor cells and have an average diameter of 200 nm, falling within this range, and can retain the surface antigens that are present on tumor cells. However, antigen uptake is not the only important metric for vaccine immunotherapy. The activation of antigen presenting cells, especially DCs, is important for the initiation or enforcement of the T cell response against malignant cells. The cytokine GM-CSF has been known to induce DC maturation and activation [25,26]. One of the earliest iterations was in the form of GVAX [9], followed by Sipuleucel-T [27], Talimogene laherparevec (T-VEC) [28], and many other approaches [29]. These approaches rely on the delivery of GM-CSF ex vivo to patient PBMCs, or the generalized production of GM-CSF into the periphery in the case of GVAX or T-VEC. Using the TMVs, DCs are presented with antigen in a formulation that is easily internalized, and results in DC maturation and inflammatory cytokine production induced by the TMV-bound GM-CSF and IL-12. As with GM-CSF, IL-12 dosage and delivery is important for the tolerance and efficacy of treatment. Frequent systemic dosages of IL-12 have been found to be toxic in human studies, while a single dose two weeks before consecutive dosing dramatically reduced the toxicity in Phase 1 studies [30]. In order to decrease the toxicity in cancer patients undergoing IL-12 therapy, new approaches such as localized delivery of IL-12 intratumorally, or antibody-cytokine fusion proteins such as NHS-IL-12, are being tested in patients [31,32,33]. In a recent first-in-human phase 1 trial, an MTD of 16.8 μg/kg of NHS-IL-12 administered subcutaneously (s.c.) was reported in lung cancer patients resulting in increased tumor-infiltrating lymphocytes and T cell receptor diversity, although no objective tumor responses were observed [34]. Delivery of DCs pulsed ex vivo with TMVs containing GPI-IL-12 may greatly decrease the potential local and systemic toxicity of the cytokine, while promoting tumor specific T cell activation and differentiation. Further, the ex vivo pulsing of DCs with TMVs containing GPI-GM-CSF can limit the off-target immunosuppressive effects of GM-CSF on other myeloid cells, such as MDSCs, which can have a negative effect on the anti-tumor immune response [35].

Immune cell infiltration into tumors is one of the biomarkers used to predict responses in patients, and a high infiltration of T cells is associated with decreased metastatic recurrence and increased survival in TNBC patients [36,37]. However, T cells are not the only important subset of immune cells that play a role in the tumor microenvironment. It has become clearer that the presence of antigen presenting cells, particularly DCs, can provide a niche for optimal T cell responses within tumors [38]. In this study we compare the T cell and DC infiltration within 4T1 tumors after treatment with the DC vaccine. The tumors from mice treated with the DC vaccine had a significant increase in the percentage of MHC-II^+^ CD11c^+^ DCs compared to PBS controls. Further, there was a significant increase in the percentage of tumor infiltrating CD4 and CD8 T cells. Overall, treatment with the DC vaccine therapeutic approach results in a beneficial change in the immune profile of 4T1 tumors, however, more remains to be known about the specific mechanisms that govern the changes in DC and T cell infiltration. Overall, our studies serve as a proof of concept for the use of TMVs with GPI-ISMs as the source of antigen and adjuvants for the development of a therapeutic DC vaccine platform. Our studies show the potential for use in cancers where there is a defined tumor associated antigen, or in metastatic cancers that lack a defined antigen such as triple negative breast cancer. 

## Data Availability

Data are available upon reasonable request to the author.

## Supplementary Materials

The following are available online at https://www.mdpi.com/article/10.3390/ijms22168377/s1. Figure S1: TMVs labeled with pHrodo only fluoresce in low pH environment. D2F2/E2 TMVs were labeled as described in methods. Unlabeled and pHrodo labeled TMVs were placedin FACS buffer at pH 7 or in FACS buffer at pH 4 with HCl. The pHrodo Deep Red fluorescence has anemission peak of 640 nm detected on the APC channel.

## References

[1] Le Gall C.M., Weiden J., Eggermont L.J., Figdor C.G. (2018). Dendritic cells in cancer immunotherapy. Nat. Mater..

[2] Kantoff P.W., Higano C.S., Shore N.D., Berger E.R., Small E.J., Penson D.F., Redfern C.H., Ferrari A.C., Dreicer R., Sims R.B. (2010). Sipuleucel-T immunotherapy for castration-resistant prostate cancer. N. Engl. J. Med..

[3] Carreno B.M., Magrini V., Becker-Hapak M., Kaabinejadian S., Hundal J., Petti A.A., Ly A., Lie W.R., Hildebrand W.H., Mardis E.R. (2015). Cancer immunotherapy. A dendritic cell vaccine increases the breadth and diversity of melanoma neoantigen-specific T cells. Science.

[4] Kavanagh B., Ko A., Venook A., Margolin K., Zeh H., Lotze M., Schillinger B., Liu W., Lu Y., Mitsky P. (2007). Vaccination of metastatic colorectal cancer patients with matured dendritic cells loaded with multiple major histocompatibility complex class I peptides. J. Immunother..

[5] Burgdorf S.K. (2010). Dendritic cell vaccination of patients with metastatic colorectal cancer. Dan. Med. Bull..

[6] Wilgenhof S., Corthals J., Heirman C., van Baren N., Lucas S., Kvistborg P., Thielemans K., Neyns B. (2016). Phase II Study of Autologous Monocyte-Derived mRNA Electroporated Dendritic Cells (TriMixDC-MEL) Plus Ipilimumab in Patients With Pretreated Advanced Melanoma. J. Clin. Oncol..

[7] Sahin U., Derhovanessian E., Miller M., Kloke B.P., Simon P., Lower M., Bukur V., Tadmor A.D., Luxemburger U., Schrors B. (2017). Personalized RNA mutanome vaccines mobilize poly-specific therapeutic immunity against cancer. Nature.

[8] Dorrie J., Schaft N., Schuler G., Schuler-Thurner B. (2020). Therapeutic Cancer Vaccination with Ex Vivo RNA-Transfected Dendritic Cells-An Update. Pharmaceutics.

[9] van den Eertwegh A.J., Versluis J., van den Berg H.P., Santegoets S.J., van Moorselaar R.J., van der Sluis T.M., Gall H.E., Harding T.C., Jooss K., Lowy I. (2012). Combined immunotherapy with granulocyte-macrophage colony-stimulating factor-transduced allogeneic prostate cancer cells and ipilimumab in patients with metastatic castration-resistant prostate cancer: A phase 1 dose-escalation trial. Lancet Oncol..

[10] Santegoets S.J., Stam A.G., Lougheed S.M., Gall H., Jooss K., Sacks N., Hege K., Lowy I., Scheper R.J., Gerritsen W.R. (2014). Myeloid derived suppressor and dendritic cell subsets are related to clinical outcome in prostate cancer patients treated with prostate GVAX and ipilimumab. J. Immunother. Cancer.

[11] Tanyi J.L., Chiang C.L., Chiffelle J., Thierry A.C., Baumgartener P., Huber F., Goepfert C., Tarussio D., Tissot S., Torigian D.A. (2021). Personalized cancer vaccine strategy elicits polyfunctional T cells and demonstrates clinical benefits in ovarian cancer. NPJ Vaccines.

[12] Chiang C.L., Kandalaft L.E., Tanyi J., Hagemann A.R., Motz G.T., Svoronos N., Montone K., Mantia-Smaldone G.M., Smith L., Nisenbaum H.L. (2013). A dendritic cell vaccine pulsed with autologous hypochlorous acid-oxidized ovarian cancer lysate primes effective broad antitumor immunity: From bench to bedside. Clin. Cancer Res..

[13] Foged C., Brodin B., Frokjaer S., Sundblad A. (2005). Particle size and surface charge affect particle uptake by human dendritic cells in an in vitro model. Int. J. Pharm..

[14] Genito C.J., Batty C.J., Bachelder E.M., Ainslie K.M. (2021). Considerations for Size, Surface Charge, Polymer Degradation, Co-Delivery, and Manufacturability in the Development of Polymeric Particle Vaccines for Infectious Diseases. Adv. Nanobiomed. Res..

[15] Patel J.M., Vartabedian V.F., Bozeman E.N., Caoyonan B.E., Srivatsan S., Pack C.D., Dey P., D’Souza M.J., Yang L., Selvaraj P. (2016). Plasma membrane vesicles decorated with glycolipid-anchored antigens and adjuvants via protein transfer as an antigen delivery platform for inhibition of tumor growth. Biomaterials.

[16] Pack C.D., Bommireddy R., Munoz L.E., Patel J.M., Bozeman E.N., Dey P., Radhakrishnan V., Vartabedian V.F., Venkat K., Ramachandiran S. (2020). Tumor membrane-based vaccine immunotherapy in combination with anti-CTLA-4 antibody confers protection against immune checkpoint resistant murine triple-negative breast cancer. Hum. Vaccin. Immunother..

[17] McHugh R.S., Nagarajan S., Wang Y.C., Sell K.W., Selvaraj P. (1999). Protein transfer of glycosyl-phosphatidylinositol-B7-1 into tumor cell membranes: A novel approach to tumor immunotherapy. Cancer Res..

[18] Bommireddy R., Munoz L.E., Kumari A., Huang L., Fan Y., Monterroza L., Pack C.D., Ramachandiran S., Reddy S.J.C., Kim J. (2020). Tumor Membrane Vesicle Vaccine Augments the Efficacy of Anti-PD1 Antibody in Immune Checkpoint Inhibitor-Resistant Squamous Cell Carcinoma Models of Head and Neck Cancer. Vaccines.

[19] Piechocki M.P., Pilon S.A., Kelly C., Wei W.Z. (2001). Degradation signals in ErbB-2 dictate proteasomal processing and immunogenicity and resist protection by cis glycine-alanine repeat. Cell Immunol..

[20] Lutz M.B., Kukutsch N., Ogilvie A.L., Rossner S., Koch F., Romani N., Schuler G. (1999). An advanced culture method for generating large quantities of highly pure dendritic cells from mouse bone marrow. J. Immunol. Methods.

[21] Danaei M., Dehghankhold M., Ataei S., Hasanzadeh Davarani F., Javanmard R., Dokhani A., Khorasani S., Mozafari M.R. (2018). Impact of Particle Size and Polydispersity Index on the Clinical Applications of Lipidic Nanocarrier Systems. Pharmaceutics.

[22] Ebrahimi-Nik H., Corwin W.L., Shcheglova T., Mohapatra A.D., Mandoiu I.I., Srivastava P.K. (2018). CD11c(+) MHCII(lo) GM-CSF-bone marrow-derived dendritic cells act as antigen donor cells and as antigen presenting cells in neoepitope-elicited tumor immunity against a mouse fibrosarcoma. Cancer Immunol. Immunother..

[23] Hassett K.J., Higgins J., Woods A., Levy B., Xia Y., Hsiao C.J., Acosta E., Almarsson O., Moore M.J., Brito L.A. (2021). Impact of lipid nanoparticle size on mRNA vaccine immunogenicity. J. Control Release.

[24] Harding C.V., Collins D.S., Slot J.W., Geuze H.J., Unanue E.R. (1991). Liposome-encapsulated antigens are processed in lysosomes, recycled, and presented to T cells. Cell.

[25] Xu Y., Zhan Y., Lew A.M., Naik S.H., Kershaw M.H. (2007). Differential development of murine dendritic cells by GM-CSF versus Flt3 ligand has implications for inflammation and trafficking. J. Immunol..

[26] Mach N., Gillessen S., Wilson S.B., Sheehan C., Mihm M., Dranoff G. (2000). Differences in dendritic cells stimulated in vivo by tumors engineered to secrete granulocyte-macrophage colony-stimulating factor or Flt3-ligand. Cancer Res..

[27] Sims R.B. (2012). Development of sipuleucel-T: Autologous cellular immunotherapy for the treatment of metastatic castrate resistant prostate cancer. Vaccine.

[28] Bommareddy P.K., Patel A., Hossain S., Kaufman H.L. (2017). Talimogene Laherparepvec (T-VEC) and Other Oncolytic Viruses for the Treatment of Melanoma. Am. J. Clin. Dermatol..

[29] Yan W.L., Shen K.Y., Tien C.Y., Chen Y.A., Liu S.J. (2017). Recent progress in GM-CSF-based cancer immunotherapy. Immunotherapy.

[30] Leonard J.P., Sherman M.L., Fisher G.L., Buchanan L.J., Larsen G., Atkins M.B., Sosman J.A., Dutcher J.P., Vogelzang N.J., Ryan J.L. (1997). Effects of single-dose interleukin-12 exposure on interleukin-12-associated toxicity and interferon-gamma production. Blood.

[31] Fallon J., Tighe R., Kradjian G., Guzman W., Bernhardt A., Neuteboom B., Lan Y., Sabzevari H., Schlom J., Greiner J.W. (2014). The immunocytokine NHS-IL12 as a potential cancer therapeutic. Oncotarget.

[32] Fallon J.K., Vandeveer A.J., Schlom J., Greiner J.W. (2017). Enhanced antitumor effects by combining an IL-12/anti-DNA fusion protein with avelumab, an anti-PD-L1 antibody. Oncotarget.

[33] Greiner J.W., Morillon Y.M., Schlom J. (2021). NHS-IL12, a Tumor-Targeting Immunocytokine. Immunotargets Ther..

[34] Strauss J., Heery C.R., Kim J.W., Jochems C., Donahue R.N., Montgomery A.S., McMahon S., Lamping E., Marte J.L., Madan R.A. (2019). First-in-Human Phase I Trial of a Tumor-Targeted Cytokine (NHS-IL12) in Subjects with Metastatic Solid Tumors. Clin. Cancer Res..

[35] Ma N., Liu Q., Hou L., Wang Y., Liu Z. (2017). MDSCs are involved in the protumorigenic potentials of GM-CSF in colitis-associated cancer. Int. J. Immunopathol. Pharmacol..

[36] Loi S., Michiels S., Salgado R., Sirtaine N., Jose V., Fumagalli D., Kellokumpu-Lehtinen P.L., Bono P., Kataja V., Desmedt C. (2014). Tumor infiltrating lymphocytes are prognostic in triple negative breast cancer and predictive for trastuzumab benefit in early breast cancer: Results from the FinHER trial. Ann. Oncol..

[37] Loi S., Sirtaine N., Piette F., Salgado R., Viale G., Van Eenoo F., Rouas G., Francis P., Crown J.P., Hitre E. (2013). Prognostic and predictive value of tumor-infiltrating lymphocytes in a phase III randomized adjuvant breast cancer trial in node-positive breast cancer comparing the addition of docetaxel to doxorubicin with doxorubicin-based chemotherapy: BIG 02-98. J. Clin. Oncol..

[38] Jansen C.S., Prokhnevska N., Master V.A., Sanda M.G., Carlisle J.W., Bilen M.A., Cardenas M., Wilkinson S., Lake R., Sowalsky A.G. (2019). An intra-tumoral niche maintains and differentiates stem-like CD8 T cells. Nature.

