# Supplementary material for: Dendritic Cells Pulsed with Cytokine-Adjuvanted Tumor Membrane Vesicles Inhibit Tumor Growth in HER2-Positive and Triple Negative Breast Cancer Models
Source: Int J Mol Sci. 2021 Aug 4;22(16):8377. doi: 10.3390/ijms22168377 (PMC8395038; doi:10.3390/ijms22168377)
Supplement: Supplementary file 1 [file ijms-22-08377-s001.zip › ijms-1289822-supplementary.pdf]

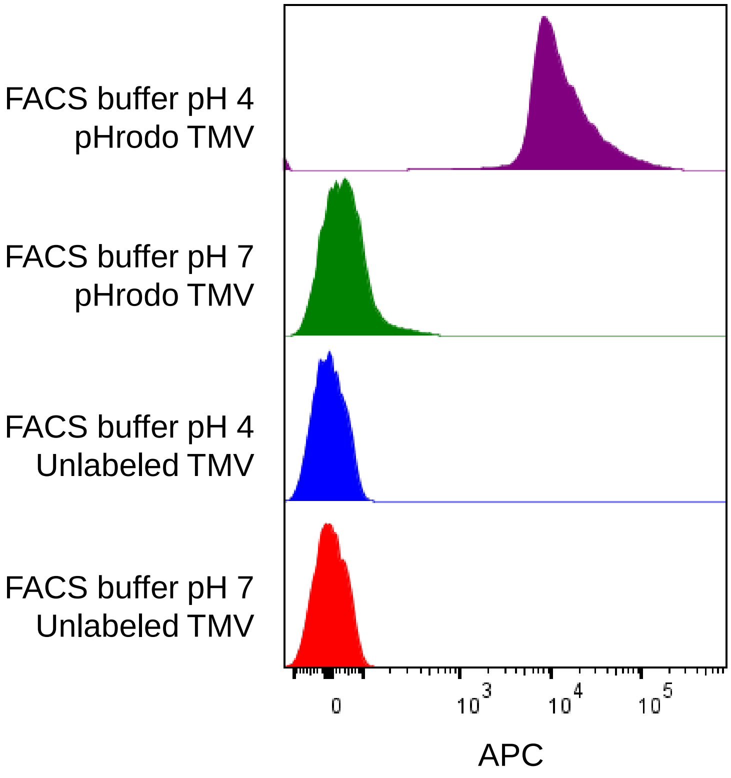

Supplementary Figure S1. TMVs labeled with pHrodo only fluoresce in low pH environment. D2F2/E2 TMVs were labeled as described in methods. Unlabeled and pHrodo labeled TMVs were placed in FACS buffer at pH 7 or in FACS buffer at pH 4 with HCl. The pHrodo Deep Red fluorescence has an emission peak of 640 nm detected on the APC channel.
